# E-Cigarette Use Among US Adults in the 2021 Behavioral Risk Factor Surveillance System Survey

**DOI:** 10.1001/jamanetworkopen.2023.40859

**Published:** 2023-11-03

**Authors:** John Erhabor, Ellen Boakye, Olufunmilayo Obisesan, Albert D. Osei, Erfan Tasdighi, Hassan Mirbolouk, Andrew P. DeFilippis, Andrew C. Stokes, Glenn A. Hirsch, Emelia J. Benjamin, Carlos J. Rodriguez, Omar El Shahawy, Rose Marie Robertson, Aruni Bhatnagar, Michael J. Blaha

**Affiliations:** 1 Johns Hopkins Ciccarone Center for Prevention of Cardiovascular Disease, Baltimore, Maryland; 2 American Heart Association Tobacco Regulation and Addiction Center, Dallas, Texas; 3 Department of Medicine, MedStar Union Memorial Hospital, Baltimore, Maryland; 4 Department of Internal Medicine, Yale School of Medicine, New Haven, Connecticut; 5 Department of Medicine, Vanderbilt University Medical Center, Nashville, Tennessee; 6 Department of Global Health, Boston University School of Public Health, Boston, Massachusetts; 7 Division of Cardiology, Department of Medicine, National Jewish Health, Denver, Colorado; 8 Cardiovascular Medicine, Boston Medical Center, Boston University Chobanian and Avedisian School of Medicine, Boston, Massachusetts; 9 Department of Epidemiology, Boston University School of Public Health, Boston, Massachusetts; 10 Albert Einstein College of Medicine, Bronx, New York, New York; 11 Department of Population Health, New York University School of Medicine, New York, New York; 12 University of Louisville School of Medicine, Louisville, Kentucky

## Abstract

**Importance:**

After the initial disruption from the COVID-19 pandemic, it is unclear how patterns of e-cigarette use in the US have changed.

**Objective:**

To examine recent patterns in current and daily e-cigarette use among US adults in 2021.

**Design, Setting, and Participants:**

This cross-sectional study used data from the 2021 Behavioral Risk Factor Surveillance System (BRFSS) database. The BRFSS is the largest national telephone-based survey of randomly sampled adults in the US. Adults aged 18 years or older, residing in 49 US states (all except Florida), the District of Columbia, and 3 US territories (Guam, Puerto Rico, and the US Virgin Islands), were included in the data set. Data analysis was performed in January 2023.

**Main Outcomes and Measures:**

The main outcome was age-adjusted prevalence of current and daily e-cigarette use overall and by participant characteristics, state, and territory. Descriptive statistical analysis was conducted, applying weights to account for population representation.

**Results:**

This study included 414 755 BRFSS participants with information on e-cigarette use. More than half of participants were women (51.3%). In terms of race and ethnicity, 0.9% of participants were American Indian or Alaska Native, 5.8% were Asian, 11.5% were Black, 17.3% were Hispanic, 0.2% were Native Hawaiian or Other Pacific Islander, 62.2% were White, 1.4% were of multiple races or ethnicities, and 0.6% were of other race or ethnicity. Individuals aged 18 to 24 years comprised 12.4% of the study population. The age-standardized prevalence of current e-cigarette use was 6.9% (95% CI, 6.7%-7.1%), with almost half of participants using e-cigarettes daily (3.2% [95% CI, 3.1%-3.4%]). Among individuals aged 18 to 24 years, there was a consistently higher prevalence of e-cigarette use, with more than 18.6% reporting current use and more than 9.0% reporting daily use. Overall, among individuals reporting current e-cigarette use, 42.2% (95% CI, 40.7%-43.7%) indicated former combustible cigarette use, 37.1% (95% CI, 35.6%-38.6%) indicated current combustible cigarette use, and 20.7% (95% CI, 19.7%-21.8%) indicated never using combustible cigarettes. Although relatively older adults (aged ≥25 years) who reported current e-cigarette use were more likely to report former or current combustible cigarette use, younger adults (aged 18-24 years) were more likely to report never using combustible cigarettes. Notably, the proportion of individuals who reported current e-cigarette use and never using combustible cigarettes was higher in the group aged 18 to 20 years (71.5% [95% CI, 66.8%-75.7%]) compared with those aged 21 to 24 years (53.0% [95% CI, 49.8%-56.1%]).

**Conclusion and Relevance:**

These findings suggest that e-cigarette use remained common during the COVID-19 pandemic, particularly among young adults aged 18 to 24 years (18.3% prevalence). Notably, 71.5% of individuals aged 18 to 20 years who reported current e-cigarette use had never used combustible cigarettes. These results underscore the rationale for the implementation and enforcement of public health policies tailored to young adults.

## Key Points

**Question:**

How have the patterns of e-cigarette use changed after the initial disruption from the COVID-19 pandemic?

**Finding:**

In this cross-sectional study of 414 755 respondents to the 2021 Behavioral Risk Factor Surveillance System survey, a high prevalence of e-cigarette use was observed among US adults, particularly among young adults aged 18 to 24 years (18%). Within the group aged 18 to 20 years, 72% of those who reported current e-cigarette use had no history of combustible cigarette use.

**Meaning:**

These findings highlight the importance of continuous surveillance of tobacco consumption patterns given their dynamic and evolving characteristics, and they underscore the potential implications for policy formulation and regulation, especially concerning young adults.

## Teaser

This cross-sectional study uses data from the 2021 Behavioral Risk Factor Surveillance System survey to assess recent patterns in current and daily e-cigarette use among US adults.

## Introduction

E-cigarettes are the second most commonly used tobacco product among US adults.^1,2^ Over the past 5 years, prevalence estimates of e-cigarette use have fluctuated across various surveys. However, these estimates have consistently remained greater than 3%, as demonstrated by data from both the Behavioral Risk Factor Surveillance System (BRFSS) and the National Health Interview Survey (NHIS).^1,3,4,5,6^ A concerning trend observed in 2 publications based on prior BRFSS data is the increasing proportion of individuals using e-cigarettes daily among those who reported use in the past 30 days over the same period. This finding suggests a potential shift from experimental to established use.^5,6^

Recent reports from the NHIS and the National Survey on Drug Use and Health (NSDUH) indicate that in 2021, the prevalence of e-cigarette use in the past 30 days was 4.5% and 6.0%, respectively.^2,7^ Across these surveys, adults aged younger than 35 years showed a notably higher prevalence of e-cigarette use. Similarly, National Youth Tobacco Survey data have consistently reported high e-cigarette use among middle and high school students for the past decade.^8^ Taken together, these findings suggest that adolescents who initiate e-cigarette use may continue into adulthood, leading to a potentially higher prevalence in young adults.

Although some studies suggest that e-cigarettes could serve as a smoking cessation aid for adults who use combustible cigarettes, their increased use among youths, young adults, and those with no prior exposure to combustible cigarettes continues to be a public health concern.^6,9,10^ The allure of and the increased use of e-cigarettes among these groups raise concerns about the potential risks of nicotine addiction, gateway effects, and long-term health implications of e-cigarette use, which have not been fully elucidated.^11,12^ Consequently, close monitoring of recent shifts in e-cigarette use within these populations is of importance.

The trajectory of e-cigarette use among US adults may have been affected by the COVID-19 pandemic. This health crisis, marked by an intensified focus on personal health and safety, may have contributed to changes in attitudes and behaviors relating to e-cigarette use.^13^ Psychological stress and isolation that may have arisen from lockdown measures also may have prompted some individuals to increasingly depend on e-cigarettes and other tobacco products as a coping mechanism.^14,15^ In addition, the COVID-19 pandemic disrupted the conduct of surveys, especially those that relied on in-person data collection. As a result, surveys such as the NHIS transitioned from in-person to online interviews.^16^ Because the BRFSS is an online-based survey, its data collection was not notably affected by the lockdown measures implemented during the COVID-19 pandemic.^17^

It is important to provide up-to-date and comprehensive data on e-cigarette use, particularly among susceptible population groups. To address this need, we used the 2021 BRFSS to examine recent patterns of e-cigarette use among US adults. Our study analyzed recent e-cigarette use patterns among US adults to monitor existing policies and guide the development of strategies to address potential health risks and improve public health.

## Methods

### Study Population, Study Sample, and Study Design

The BRFSS is an extensive, nationally representative data set of health-related telephone survey data of noninstitutionalized US adults aged 18 years or older. The survey uses iterative proportional fitting for weighting, adjusting for demographic differences, noncoverage, and nonresponse to ensure data representativeness.^18^ For this cross-sectional study, we used data from the 2021 BRFSS survey, with a survey response rate of 44.0%.^19^ Verbal consent was obtained during initial contact and screening. In 2021, 50 states, the District of Columbia, Guam, Puerto Rico, and the US Virgin Islands collected data via landline and cellular telephones.^17^ However, Florida did not collect data for sufficient months in 2021 to meet the inclusion criteria for the annual aggregate data set.^17^ We analyzed data from 414 755 participants (94.6% of the surveyed population) who provided information on e-cigarette use. Johns Hopkins School of Medicine deemed our study exempt from review because deidentified publicly available BRFSS data were used consistent with the Common Rule. This study followed the Strengthening the Reporting of Observational Studies in Epidemiology (STROBE) reporting guideline.

### Assessment of E-Cigarette and Combustible Cigarette Use

E-cigarette use was assessed with this question: “Do you now use e-cigarettes or other electronic vaping products every day, some days, or not at all?” Participants who responded “every day” or “some days” were considered to be currently using e-cigarettes. Individuals who were currently using e-cigarettes and responded “every day” were classified as using e-cigarettes daily.

Combustible cigarette use was determined using 2 questions: “Have you smoked at least 100 cigarettes in your entire life?” and “Do you now smoke cigarettes every day, some days, or not at all?” Respondents answering “yes” to the first question but “no” to the second were classified as having formerly used combustible cigarettes. If they answered “yes” to both questions, they were categorized as currently using combustible cigarettes. A “no” response to the first question indicated they had never used combustible cigarettes.

### Other Study Measures

Sociodemographic characteristics included age, sex, race and ethnicity, sexual orientation (heterosexual, lesbian or gay, or bisexual), transgender identity (yes or no), marital status (married, divorced, widowed, single, or member of an unmarried couple), education level (less than high school, high school or some college, or college graduate), employment status (employed, unemployed, student, or retired), area of residence (rural or urban), and pregnant (yes or no). Race and ethnicity were reported as American Indian or Alaska Native, Hispanic, Native Hawaiian or Other Pacific Islander, non-Hispanic Asian (hereinafter, Asian), non-Hispanic Black (hereinafter, Black), non-Hispanic White (hereinafter, White), multiple races or ethnicities, or other race or ethnicity (specific groups in the last category were not itemized in the 2021 BRFSS data set). Household income level was based on the 2021 federal poverty line.^20^ Weight and height were self-reported; body mass index was calculated as weight in kilograms divided by height in meters squared.

Chronic health conditions assessed included cardiovascular disease, diabetes, cancer (excluding skin cancer), chronic obstructive pulmonary disease (yes or no), and depression (yes or no). Cardiovascular disease was defined as a composite of myocardial infarction, coronary heart disease, or stroke. All measures were self-reported.

### Statistical Analysis

We summarized participant sociodemographics and chronic health conditions using proportions for the entire sample and for those reporting current and daily e-cigarette use. We calculated the age-standardized prevalence of current and daily e-cigarette use overall, within subgroups including combustible cigarette use categories (never, former, or current), and across age groups.

To understand tobacco use patterns, we analyzed the prevalence of combustible cigarette use among those reporting current and daily e-cigarette use. In addition, we explored different patterns of current e-cigarette and combustible use, including sole e-cigarette use, dual use, and exclusive combustible cigarette use. Finally, we estimated the age-standardized prevalence of e-cigarette use by state, allowing for comparisons while adjusting for variations in age distribution.

Statistical analysis was performed using Stata, version 16 (StataCorp LLC). We calculated weighted prevalence estimates using the BRFSS analytic recommendations.^21^ We used the BRFSS complex sampling design and participant weights for population-representative estimates. The weighted sample sizes ensured that our results mirrored population trends. Age standardization used the 2010 US Census for groups aged 18 to 24 through 55 to 59 years and 60 years or older, aiding in identifying age-standardized prevalence of e-cigarette use by participant attributes. We employed the survey command svy to account for the complex weighting method used by the BRFSS. Statistical significance was set at a 2-sided *P* < .05. Data analysis was performed in January 2023.

## Results

### Study Population

This study included 414 755 adults; 48.7% were men and 51.3% were women. Individuals aged 18 to 24 years comprised 12.4% of the study population. A total of 0.9% of participants identified as American Indian or Alaska Native, 5.8% as Asian, 11.5% as Black, 17.3% as Hispanic, 0.2% as Native Hawaiian or Other Pacific Islander, 62.2% as White, 1.4% as multiple races or ethnicities, and 0.6% as other race or ethnicity (Table 1). In the overall study sample, the proportion of individuals who reported current and daily e-cigarette use was higher among male, bisexual, transgender, and single individuals (Table 1).

**Table 1.** Participant Characteristics Overall and Among Those Who Reported Current and Daily E-Cigarette Use, 2021 Behavioral Risk Factor Surveillance System

Variable	No. (weighted %) of participants		
	Total (N = 414 755)	Current e-cigarette use (n = 19 346)	Daily e-cigarette use (n = 9050)
Sex			
Male	192 435 (48.7)	10 563 (57.0)	5002 (57.3)
Female	222 328 (51.3)	8783 (43.0)	4735 (42.7)
Age, y			
18-20	9774 (5.6)	1948 (15.3)	896 (14.0)
21-24	14 824 (6.8)	2828 (19.2)	1341 (20.0)
25-29	20 397 (7.8)	2573 (15.9)	1235 (15.7)
30-34	24 206 (9.8)	2266 (13.6)	1093 (13.5)
35-39	26 841 (7.8)	1961 (8.4)	899 (8.3)
40-44	27 661 (8.6)	1563 (7.9)	777 (8.5)
45-49	26 840 (6.5)	1191 (4.4)	556 (4.4)
50-54	32 128 (8.2)	1125 (4.6)	531 (4.8)
55-59	35 980 (7.8)	1067 (3.8)	483 (4.0)
≥60	188 084 (31.1)	2606 (7.0)	1145 (6.8)
Race and ethnicity			
American Indian or Alaska Native	6838 (0.9)	411 (1.2)	151 (0.8)
Asian, non-Hispanic	10 514 (5.8)	521 (4.9)	212 (3.2)
Black, non-Hispanic	30 150 (11.5)	1085 (9.1)	356 (6.7)
Hispanic	35 929 (17.3)	1930 (15.2)	751 (11.8)
Native Hawaiian or Other Pacific Islander	1846 (0.2)	4.9 (3.3-7.2)	50.3 (33.4-67.1)
White, non-Hispanic	307 582 (62.2)	13 862 (66.2)	6884 (74.1)
Multiple	8760 (1.4)	778 (2.4)	358 (2.4)
Other^a^	3599 (0.6)	185 (0.5)	77 (0.5)
Sexual orientation			
Heterosexual	214 530 (92.4)	9212 (82.3)	4373 (80.1)
Lesbian or gay	4303 (2.0)	398 (3.9)	202 (4.6)
Bisexual	6569 (3.7)	1024 (10.6)	493 (11.6)
Other	3388 (1.8)	313 (3.3)	174 (3.7)
Transgender			
No	231 762 (99.2)	10 860 (98.3)	5153 (97.8)
Yes	1453 (0.8)	180 (1.7)	93 (2.2)
BMI			
<18.5	6111 (1.9)	561 (3.7)	296 (4.5)
≥18.5 to <25	112 883 (30.6)	6322 (36.7)	2889 (35.0)
≥25 to <30	135 644 (34.4)	5796 (30.4)	2641 (29.9)
≥30	128 782 (33.1)	5767 (29.2)	2810 (30.6)
Marital status			
Married	215 226 (50.4)	5728 (27.3)	2859 (29.8)
Divorced or separated	60 789 (12.8)	3129 (12.4)	1452 (12.1)
Widowed	45 030 (0.9)	776 (2.4)	321 (2.6)
Single	65 309 (24.7)	7655 (47.6)	3382 (43.6)
Highest education level			
Less than high school	24 227 (11.9)	1383 (11.7)	585 (10.8)
High school or some college	218 749 (57.8)	13 360 (72.6)	6504 (75.1)
College graduate	169 978 (30.3)	4543 (15.7)	1937 (14.0)
Income, poverty line, %			
<100	34 517 (11.0)	2444 (12.8)	957 (9.6)
100-200	67 367 (17.2)	4353 (21.8)	2086 (22.0)
>200	308 410 (71.8)	12 345 (65.4)	5911 (68.4)
Employment status			
Employed	212 866 (57.2)	12 410 (66.1)	6134 (70.0)
Unemployed	61 437 (17.9)	3957 (19.9)	1707 (18.6)
Student	10 217 (5.1)	1216 (9.4)	452 (6.7)
Retired	126 605 (19.9)	1595 (4.7)	684 (4.7)
Area of residence			
Rural	59 348 (6.5)	2203 (6.3)	1018 (6.6)
Urban	348 554 (93.5)	16 913 (93.7)	7937 (93.4)
Combustible cigarette use			
Never	245 395 (62.9)	5322 (32.8)	1848 (24.8)
Former	112 749 (23.7)	7310 (36.1)	4846 (50.9)
Current	53 547 (13.4)	6552 (31.1)	2282 (24.4)
Pregnant			
No	75 227 (96.6)	5984 (98.1)	2798 (97.2)
Yes	2297 (3.4)	91 (1.9)	47 (2.8)
Chronic health condition			
CVD^b^			
No	365 624 (91.6)	17 868 (95.0)	8432 (94.8)
Yes	44 714 (8.4)	1284 (5.0)	553 (5.2)
Cancer			
No	373 099 (92.9)	18 257 (96.4)	8557 (96.2)
Yes	40 507 (7.1)	1023 (3.6)	462 (3.8)
COPD			
No	380 259 (93.5)	17 451 (93.1)	8216 (93.3)
Yes	32 636 (6.5)	1786 (6.9)	793 (6.7)
Asthma			
No	354 658 (85.3)	15 336 (79.0)	7208 (78.8)
Yes	58 564 (14.7)	3903 (21.0)	1790 (21.2)
Depression			
No	330 584 (80.1)	11 814 (62.4)	5279 (59.7)
Yes	81 959 (19.9)	7378 (37.6)	3698 (40.3)

Abbreviations: BMI, body mass index (calculated as weight in kilograms divided by height in meters squared); COPD, chronic obstructive pulmonary disease; CVD, cardiovascular disease.

^a^ Specific groups within the other race and ethnicity category were not itemized in the 2021 Behavioral Risk Factor Surveillance System data set.

^b^ Composite of myocardial infarction, coronary heart disease, and/or stroke.

### Patterns of E-Cigarette Use Among US Adults Overall and by Sociodemographic Characteristics

The age-standardized prevalence of current and daily e-cigarette use was 6.9% (95% CI, 6.7%-7.1%; weighted sample approximately 15 million) and 3.2% (95% CI, 3.1%-3.4%; weighted sample approximately 7 million), respectively (Table 2). Among individuals who reported current e-cigarette use, the proportion of daily use, as a measure of established use and possible nicotine addiction, was 46.6% (95% CI, 45.3%-48.0%) (Table 2 and eTable 1 in Supplement 1).

**Table 2.** Age-Standardized Weighted Prevalence of E-Cigarette Use (Current and Daily) Among US Adults, 2021 Behavioral Risk Factor Surveillance System

Variable	Age-standardized weighted prevalence, % (95% CI)		Daily-to-current use ratio
	Current e-cigarette use	Daily e-cigarette use	
Total population (N = 414 755)	6.9 (6.7-7.1)	3.2 (3.1-3.4)	46.6 (45.3-48.0)
Sex			
Male	7.8 (7.6-8.1)	3.7 (3.5-3.9)	46.9 (45.1-48.7)
Female	6.0 (5.7-6.2)	2.8 (2.6-2.9)	46.3 (44.1-48.4)
Race and ethnicity			
American Indian or Alaska Native	8.7 (7.4-10.1)	2.9 (2.3-3.7)	33.6 (26.8-41.2)
Asian, non-Hispanic	4.7 (3.9-5.6)	1.5 (1.1-2.0)	30.3 (23.4-38.3)
Black, non-Hispanic	5.5 (5.0-6.0)	1.9 (1.6-2.2)	34.3 (30.0-38.9)
Hispanic	4.6 (4.3-5.0)	1.7 (1.5-1.9)	36.2 (32.0-40.5)
Native Hawaiian or Other Pacific Islander	10.7 (7.8-14.6)	4.9 (3.3-7.2)	50.3 (33.4-67.1)
White, non-Hispanic	8.2 (8.0-8.5)	4.3 (4.1-4.5)	52.1 (50.6-53.6)
Multiple	9.5 (8.2-10.9)	4.2 (3.5-5.1)	45.5 (38.9-52.3)
Other^a^	6.9 (5.4-8.8)	2.8 (2.0-3.9)	42.4 (30.9-54.7)
Sexual orientation			
Heterosexual	6.8 (6.6-7.1)	3.2 (3.0-3.3)	46.1 (44.2-48.1)
Lesbian or gay	10.7 (9.1-12.6)	5.7 (4.4-7.5)	55.8 (46.4-64.7)
Bisexual	12.2 (11.0-13.7)	6.5 (5.4-7.8)	52.0 (46.8-57.1)
Other	10.5 (8.4-13.2)	5.9 (4.0-8.6)	53.5 (42.9-63.9)
Transgender			
No	7.1 (6.9-7.4)	3.4 (3.2-3.5)	47.1 (45.3-48.9)
Yes	12.3 (9.3-16.3)	7.2 (4.6-11.1)	61.5 (48.1-73.3)
BMI			
<18.5	9.8 (8.3-11.5)	4.9 (4.0-5.9)	56.6 (48.4-64.4)
≥18.5 to <25	7.2 (6.9-7.6)	3.2 (3.0-3.5)	44.4 (41.9-46.8)
≥25 to <30	7.1 (6.7-7.4)	3.2 (3.0-3.5)	45.8 (43.4-48.3)
≥30	7.0 (6.7-7.4)	3.4 (3.2-3.6)	48.7 (46.3-51.1)
Marital status			
Married	5.3 (4.9-5.7)	2.8 (2.5-3.1)	51.0 (48.4-53.5)
Divorced or separated	10.0 (8.6-11.5)	4.5 (3.9-5.3)	45.6 (42.2-49.0)
Widowed	11.8 (8.7-15.8)	7.8 (5.1-11.7)	50.4 (42.0-58.8)
Single	7.6 (7.2-8.0)	3.2 (2.9-3.4)	42.7 (40.7-44.8)
Highest education level			
Less than high school	7.3 (6.7-8.0)	3.2 (2.7-3.7)	43.2 (38.6-48.0)
High school or some college	8.3 (8.0-8.5)	4.0 (3.8-4.2)	48.3 (46.7-49.9)
College graduate	4.2 (4.0-4.5)	1.7 (1.5-1.8)	41.6 (39.1-44.3)
Income, poverty line, %			
<100	7.1 (6.6-7.7)	2.5 (2.2-2.8)	35.0 (31.4-38.9)
100-200	8.3 (7.9-8.8)	3.9 (3.6-4.2)	46.9 (44.3-49.6)
>200	7.7 (6.6-9.0)	3.2 (2.5-4.1)	48.7 (47.0-50.5)
Employment status			
Employed	7.2 (7.0-7.4)	3.6 (3.4-3.7)	49.4 (47.7-51.1)
Unemployed	8.0 (7.5-8.5)	3.5 (3.1-3.9)	43.6 (40.6-46.7)
Student	9.1 (6.6-12.4)	4.2 (2.4-7.3)	33.6 (29.4-38.1)
Retired	13.9 (9.3-20.3)	9.4 (5.0-16.8)	46.8 (40.8-53.0)
Area of residence			
Rural	7.9 (7.2-8.7)	3.9 (3.3-4.5)	48.8 (44.2-53.4)
Urban	6.9 (6.7-7.1)	3.2 (3.1-3.4)	46.5 (45.1-48.0)
Combustible cigarette use			
Never	2.9 (2.8-3.1)	1.0 (0.9-1.1)	35.2 (32.9-37.7)
Former	17.2 (16.5-18.0)	11.6 (10.9-12.3)	65.8 (63.4-68.1)
Current	17.9 (17.1-18.7)	6.9 (6.3-7.4)	36.5 (34.2-38.8)
Pregnant			
No	8.8 (8.4-9.3)	4.0 (3.8-4.3)	45.6 (43.1-48.1)
Yes	5.4 (3.1-9.2)	2.8 (1.6-4.7)	67.1 (50.7-80.2)
Chronic health condition			
CVD^b^			
No	6.8 (6.6-7.0)	3.2 (3.1-3.3)	46.7 (45.3-48.1)
Yes	10.7 (8.9-12.9)	5.5 (3.9-7.6)	48.3 (42.4-54.3)
Cancer			
No	6.9 (6.7-7.1)	3.2 (3.1-3.3)	46.5 (45.1-47.9)
Yes	9.8 (7.9-12.2)	5.8 (4.0-8.3)	49.4 (43.2-55.7)
COPD			
No	6.7 (6.5-6.9)	3.1 (3.0-3.3)	46.8 (45.4-48.3)
Yes	11.7 (10.5-12.9)	5.1 (4.4-6.0)	45.1 (40.8-49.4)
Asthma			
No	6.5 (6.4-6.7)	3.0 (2.9-3.2)	46.5 (44.9-48.0)
Yes	8.8 (8.3-9.3)	4.1 (3.8-4.5)	47.1 (44.3-50.0)
Depression			
No	5.6 (5.4-5.8)	2.5 (2.4-2.6)	44.6 (42.9-46.3)
Yes	11.7 (11.2-12.2)	5.8 (5.5-6.2)	50.1 (47.8-52.4)

Abbreviations: BMI, body mass index (calculated as weight in kilograms divided by height in meters squared); COPD, chronic obstructive pulmonary disease; CVD, cardiovascular disease.

^a^ Specific groups within the other race and ethnicity category were not itemized in the 2021 Behavioral Risk Factor Surveillance System data set.

^b^ Composite of myocardial infarction, coronary heart disease, and/or stroke.

Compared with heterosexual individuals, persons who identified as bisexual had a higher prevalence of current e-cigarette use (12.2% [95% CI, 11.0%-13.7%] vs 6.8% [95% CI, 6.6%-7.1%]) and daily e-cigarette use (6.5% [95% CI, 5.4%-7.8%] vs 3.2% [95% CI, 3.0%-3.3%]). Similarly, compared with cisgender individuals, those who identified as transgender reported a higher prevalence of current e-cigarette use (12.3% [95% CI, 9.3%-16.3%] vs 7.1% [95% CI, 6.9%-7.4%]) and daily e-cigarette use (7.2% [9.5% CI, 4.6%-11.1%] vs 3.4% [95% CI, 3.2%-3.5%]). Compared with individuals without the respective comorbid condition, the prevalence of current e-cigarette use was higher among those with cardiovascular disease (10.7% [95% CI, 8.9%-12.9%] vs 6.8% [95% CI, 6.6%-7.0%]), cancer (9.8% [95% CI, 7.9%-12.2%] vs 6.9% [95% CI, 6.7%-7.1%]), asthma (8.8% [95% CI, 8.3%-9.3%] vs 6.5% [95% CI, 6.4%-6.7%]), and depression (11.7% [95% CI, 11.2%-12.2%] vs 5.6% [95% CI, 5.4%-5.8%]) (Table 2).

Among individuals who reported current e-cigarette use (daily-to-current use ratio), the proportion who used e-cigarettes daily was considerably higher among non-Hispanic White, lesbian or gay, bisexual, and transgender individuals, as well as among those who formerly smoked combustible cigarettes, compared with their respective comparison groups (Table 2).

The age-standardized prevalence of current e-cigarette use among individuals who reported never using combustible cigarettes was 2.9% (95% CI, 2.8%-3.1%). The prevalence was higher among individuals who reported former combustible cigarette use, at 17.2% (95% CI, 16.5%-18.0%), and current combustible cigarette use, at 17.9% (95% CI, 17.1%-18.7%). The age-standardized prevalence of daily e-cigarette use by smoking status showed similar patterns (Table 2).

Table 3 demonstrates the prevalence of current and daily e-cigarette use across age groups and by combustible cigarette use. The prevalence of current e-cigarette use decreased with increasing age and was highest among young adults aged 18 to 20 years (18.1% [95% CI, 16.6%-19.6%]) and 21 to 24 years (18.6% [95% CI, 17.5%-19.7%]) (Figure and Table 3).

**Table 3.** Weighted Prevalence of E-Cigarette Use (Current and Daily) by Combustible Cigarette Use (Never, Former, and Current) Across Age Groups, 2021 Behavioral Risk Factor Surveillance System

Age group, y	Weighted prevalence, % (95% CI)							
	Total population		Never combustible cigarette use		Former combustible cigarette use		Current combustible cigarette use	
	Current	Daily	Current	Daily	Current	Daily	Current	Daily
18-20	18.1 (16.6-19.6)	7.7 (6.9-8.7)	13.9 (12.7-15.3)	5.5 (4.8-6.4)	63.1 (52.3-72.7)	36.0 (26.7-46.6)	67.4 (59.7-74.4)	29.5 (23.6-36.2)
21-24	18.6 (17.5-19.7)	9.0 (8.2-9.8)	11.8 (10.9-12.9)	4.5 (3.9-5.2)	53.8 (49.0-58.5)	39.8 (35.1-44.6)	45.3 (40.6-50.1)	20.2 (17.0-23.9)
25-29	13.4 (12.5-14.3)	6.2 (5.6-6.8)	5.9 (5.2-6.7)	1.6 (1.3-1.9)	33.7 (30.6-36.9)	21.8 (19.4-24.4)	30.3 (27.0-33.8)	13.1 (10.6-16.1)
30-34	9.1 (8.5-9.8)	4.2 (3.8-4.6)	2.6 (2.2-3.1)	0.8 (0.6-1.1)	22.1 (20.1-24.2)	13.8 (12.2-15.4)	19.2 (17.1-21.4)	6.4 (5.4-7.7)
35-39	7.1 (6.5-7.6)	3.2 (2.9-3.6)	1.6 (1.2-2.1)	0.4 (0.3-0.6)	15.1 (13.5-16.7)	10.0 (8.7-11.4)	16.5 (14.6-18.5)	5.0 (3.8-6.5)
40-44	6.0 (5.5-6.6)	3.0 (2.6-3.5)	1.0 (0.7-1.4)	0.4 (0.2-0.7)	12.4 (10.9-14.1)	8.5 (7.3-9.9)	13.8 (12.1-15.8)	4.3 (3.2-5.8)
45-49	4.5 (4.0-5.0)	2.1 (1.8-2.4)	0.9 (0.6-1.2)	0.2 (0.1-0.4)	8.4 (7.3-9.7)	6.2 (5.2-7.3)	12.7 (10.7-15.0)	3.5 (2.5-4.8)
50-54	3.7 (3.3-4.1)	1.8 (1.5-2.2)	0.5 (0.3-0.7)	0.1 (0.1-0.2)	8.0 (6.6-9.5)	5.5 (4.3-6.9)	10.1 (8.6-11.8)	3.4 (2.5-4.7)
55-59	3.2 (2.8-3.6)	1.6 (1.3-1.9)	0.4 (0.3-0.6)	0.1 (0.1-0.2)	6.3 (5.3-7.4)	4.0 (3.2-5.1)	8.2 (6.9-9.6)	2.8 (2.1-3.9)
≥60	1.5 (1.4-1.6)	0.8 (0.7-0.9)	0.4 (0.3-0.5)	0.1 (0.2-0.4)	2.0 (1.7-2.2)	1.1 (1.0-1.3)	5.2 (4.6-5.9)	1.8 (1.4-3.9)

**Figure.**
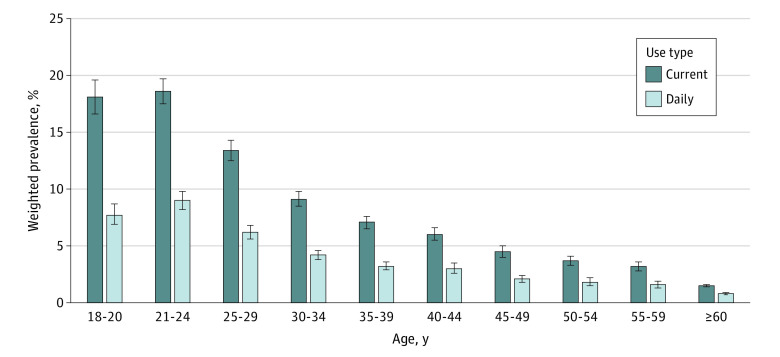
Weighted Prevalence of E-Cigarette Use Among US Adults, 2021 Behavioral Risk Factor Surveillance System Error bars indicate 95% CIs.

### Distribution of Combustible Cigarette Use Among Individuals Who Reported Current and Daily E-Cigarette Use

Among individuals who reported current e-cigarette use, 20.7% (95% CI, 19.7%-21.8%) reported never using combustible cigarettes, 42.2% (95% CI, 40.7%-43.7%) reported former combustible cigarette use, and 37.1% (95% CI, 35.6%-38.6%) reported current combustible cigarette use (Table 4). These proportions varied widely by age group. Notably, the proportion who reported never using combustible cigarettes among those who reported current e-cigarette use was highest among individuals aged 18 to 20 years (71.5% [95% CI, 66.8%-75.7%]), followed by individuals aged 21 to 24 years (53.0% [95% CI, 49.8%-56.1%]) (Table 4 and eFigure 1 in Supplement 1). Similarly, the proportion of individuals reporting never using combustible cigarettes among those who reported daily e-cigarette use was highest among young adults aged 18 to 20 years (66.5% [95% CI, 61.2%-71.4%]) (Table 4).

**Table 4.** Distribution of Combustible Cigarette Use (Never, Former, and Current) Among Individuals Who Use E-Cigarettes (Current and Daily) Across Age Groups, 2021 Behavioral Risk Factor Surveillance System

Age group, y	Weighted prevalence of combustible cigarette use, % (95% CI)		
	Never combustible cigarette use	Former combustible cigarette use	Current combustible cigarette use
**Current e-cigarette use**			
Total population	20.7 (19.7-21.8)	42.2 (40.7-43.7)	37.1 (35.6-38.6)
18-20	71.5 (66.8-75.7)	13.2 (9.5-18.2)	15.3 (12.9-18.1)
21-24	53.0 (49.8-56.1)	26.0 (23.2-29.0)	21.0 (18.6-23.6)
25-29	32.0 (28.8-35.4)	37.6 (34.2-41.1)	30.4 (27.0-34.0)
30-34	18.5 (15.8-21.7)	45.9 (42.4-49.6)	35.5 (32.1-39.1)
35-39	14.0 (10.9-17.8)	44.6 (40.6-48.7)	41.4 (37.3-45.5)
40-44	10.0 (7.3-13.6)	49.0 (44.3-53.8)	41.0 (36.4-45.7)
45-49	12.0 (9.0-15.8)	41.3 (36.2-46.5)	46.7 (41.3-52.3)
50-54	8.1 (5.6-11.7)	47.5 (41.6-53.5)	44.4 (38.7-50.2)
55-59	8.1 (6.0-10.7)	49.0 (43.3-54.6)	43.0 (37.5-48.7)
≥60	14.5 (12.0-17.5)	46.9 (42.9-51.0)	38.6 (34.6-42.6)
**Daily e-cigarette use**			
Total population	14.7 (13.6-16.0)	58.1 (55.8-60.4)	27.2 (25.0-29.4)
18-20	66.5 (61.2-71.4)	17.8 (14.0-22.2)	15.7 (12.4-19.7)
21-24	41.2 (36.6-45.9)	39.6 (35.0-44.4)	19.3 (16.2-22.8)
25-29	18.6 (15.3-22.4)	52.9 (47.8-57.9)	28.5 (23.7-33.9)
30-34	12.4 (9.4-16.2)	61.9 (57.1-66.4)	25.7 (21.9-30.0)
35-39	8.5 (6.0-11.8)	64.2 (57.9-70.0)	27.4 (21.8-33.8)
40-44	7.8 (4.4-13.4)	66.7 (59.5-73.2)	25.5 (19.7-32.4)
45-49	7.4 (4.3-12.5)	65.2 (57.1-72.5)	27.3 (20.5-35.5)
50-54	4.1 (2.5-6.6)	65.8 (56.9-73.8)	30.1 (22.5-39.1)
55-59	4.8 (2.6-8.7)	64.5 (55.7-72.5)	30.7 (23.1-39.5)
≥60	9.5 (6.8-13.3)	60.2 (53.8-66.3)	30.2 (24.4-36.8)

### Patterns of E-Cigarette and Combustible Cigarette Use

The prevalence of sole e-cigarette, exclusive combustible cigarette, and dual e-cigarette and combustible cigarette use was 4.7% (95% CI, 4.6%-4.9%), 11.7% (95% CI, 11.4%-11.9%), and 2.2% (95% CI, 2.1%-2.3%), respectively. The prevalence differed across age groups, with a higher prevalence of sole e-cigarette use among younger age groups (18-20 years: 15.2% [95% CI, 13.9%-16.7%]; and 21-24 years: 14.6% [95% CI, 13.6%-15.6%]) (eTable 2 and eFigure 2 in Supplement 1). Similarly, the prevalence of dual e-cigarette and combustible cigarette use was higher in younger age groups, with the highest prevalence seen among individuals aged 25 to 29 years (4.0% [95% CI, 3.5%-4.6%]), followed by individuals aged 21 to 24 years (3.9% [95% CI, 3.4%-4.4%]). In contrast, the prevalence of exclusive combustible cigarette use was higher among the older age groups aged 55 to 59 years (15.5% [95% CI, 14.7%-16.3%]) compared with those aged 18 to 20 years (1.3% [95% CI, 1.0%-1.8%]) (eTables 2 and 3 in Supplement 1).

### Prevalence of E-Cigarette Use by State

eTable 3 and eFigure 3 in Supplement 1 show the state-specific age-standardized prevalence of e-cigarette use. Southern, western, and midwestern states generally had a higher prevalence of current e-cigarette use compared with other states, except for California (5.2% [95% CI, 4.5%-5.9%]) and Minnesota (5.7% [95% CI, 5.2%-6.2%]). Northeastern states generally had a lower prevalence of current e-cigarette use, except for Delaware (6.1% [95% CI, 5.1%-7.4%]), New Jersey (6.0% [95% CI, 5.2%-6.8%]), Pennsylvania (6.1% [95% CI, 5.3%-7.0%]), and Rhode Island (6.2% [95% CI, 5.1%-7.4%]). In the US territories, the prevalence varied widely from 2.0% (95% CI, 1.5%-2.7%) in Puerto Rico to 11.1% in Guam (95% CI, 8.8%-14.0%).

## Discussion

In this cross-sectional study using a large, nationally representative sample of the US population, we report the prevalence and distribution of e-cigarette use among adults aged 18 years or older in 2021, providing national and state-level prevalence estimates. Overall, 6.9% of participants reported current e-cigarette use, whereas 3.2% reported daily e-cigarette use. Among individuals who reported current e-cigarette use, almost half (46.6%) reported daily e-cigarette use. The prevalence of current e-cigarette use was highest among young adults aged 18 to 20 and 21 to 24 years, with 71.5% of the former group having no history of combustible cigarette use. E-cigarette use was more prevalent among men; persons who identified as lesbian, gay, bisexual, or transgender; individuals who lived in rural areas; and those with a chronic health condition. Similar to prior studies, state-level prevalence estimates remained heterogenous, with the southern and western states generally having a higher prevalence of e-cigarette use compared with other regions.^22^

The age-standardized prevalence estimates of e-cigarette use from this study differed from that reported in the 2021 NHIS. According to the NHIS data, the prevalence of e-cigarette use among adults aged 18 years or older was 4.5%.^23^ This variation in estimates may likely be attributed to differences in survey methodologies, sampling variability, and timing of data collection between data sets. Whereas the BRFSS is solely an online survey, the NHIS involves in-person interviews and physical examinations.^16,24^ However, the COVID-19 pandemic prompted the NHIS to shift from in-person to online interviews. This sudden methodological change, along with sampling variability and timing, might explain the discrepancies in e-cigarette use prevalence estimates.^25^

Our data suggest that the prevalence of current e-cigarette use is higher than that reported in prior years. Based on BRFSS data, the prevalence of current e-cigarette use varied between 4.6% to 5.4% between 2016 and 2018.^5,6^ The higher prevalence of e-cigarette use observed in 2021 may have been due to changes that occurred during the pandemic, such as increased online sales, which facilitated easy accessibility and concomitant stockpiling.^26,27^ Moreover, the heightened psychosocial stress experienced during the pandemic may have resulted in more individuals turning to e-cigarettes as a coping mechanism.^28,29^

We observed a high proportion (71.5%) of individuals aged 18 to 20 years who reported current e-cigarette use without concurrent use of combustible cigarettes. This proportion is numerically higher than those observed in prior BRFSS years (63.3% in 2017, 66.1% in 2018, and 70.4% in 2020).^5,6^ Perceived reduced harm, easy access, and social preference for e-cigarettes over other tobacco products may explain the high e-cigarette prevalence among young adults.^30,31,32,33^ Also, with nearly one-half of individuals (46.6%) currently using e-cigarettes reporting daily use, there is an implication of a shift from experimental to established use. This observation aligns with patterns observed in prior BRFSS years (34.5% in 2017, 37.3% in 2018, and 44.4% in 2020),^5,6^ albeit with numerically higher findings in our study.

Daily e-cigarette use is linked to nicotine dependence and initiating combustible smoking in tobacco-naive adolescents^34,35^ while also increasing quit rates and reducing cigarette use among established smokers.^10,36^ The dual nature of the effects of e-cigarette use, influencing both initiation and cessation of combustible cigarettes, may help contextualize the importance of the limited overall total population-wide prevalence (<1%) yet substantial number of young adults using e-cigarettes daily without prior combustible cigarette use. It is, however, conceivable that in the absence of e-cigarettes, some young adults may have taken up or continued to use combustible cigarettes.

The observed variation in e-cigarette use prevalence across different states might potentially stem from a range of state-specific factors. Such factors may encompass the timing and response to the COVID-19 pandemic, socioeconomic conditions, stringency of tobacco regulatory policies, and the degree to which excise taxes on e-cigarette devices are enforced in these jurisdictions.^37^ These aspects are not isolated; they interact and may influence each other, potentially accounting for the heterogeneity in the prevalence rates across states.

There seem to be many implications of our data for policy and public health, particularly for those aged 18 to 20 years. Some researchers have viewed data such as ours as a reflection of mental health, considering the interplay of stress and substance use, and as a call for tighter regulation of existing policies, such as Tobacco 21 legislation and the e-cigarette flavor ban, considering the high prevalence of e-cigarette use among young adults.^29,38^ Others have suggested that tighter control of online purchases may limit e-cigarette use among young adults.^26^ Furthermore, our study highlights the importance of collecting and analyzing data continuously, as they play a vital role in monitoring epidemiologic shifts and informing policies.^39^ These data should be consistently repeated and compared across surveys to ensure accurate and up-to-date information.^39^

### Limitations

This study has certain limitations. First, it relied on self-reported data, which introduces the potential for misclassification or recall bias. In addition, social desirability and recall bias may have resulted in underreporting of both e-cigarette use and smoking status. It is important to note that these data provide a snapshot of e-cigarette use specifically in 2021, and assessing the overall impact of the entire COVID-19 pandemic on e-cigarette use presents challenges. Although COVID-19 affected our findings, its exact association with the prevalence of e-cigarette use is unclear. Future studies should assess pandemic-specific factors, such as lockdown effects on e-cigarette availability and use. Longitudinal data during the pandemic can provide insights into evolving behaviors.

## Conclusions

This cross-sectional study highlights a high prevalence of e-cigarette use among adults in the US, particularly among young adults, in 2021. A striking finding is that 71.5% of those aged 18 to 20 years who reported e-cigarette use had no prior history of combustible cigarette use—this number is numerically higher compared with prior BRFSS data. Another notable observation was the high proportion of daily use among persons who reported current e-cigarette use, indicating a possible transition toward established use and potential nicotine dependence. These findings are of value to the tobacco regulatory science community and to policy makers, and they underscore the rationale for the implementation and enforcement of public health policies tailored to young adults.

## Notes

Supplement 1. **eTable 1.** Weighted Sample Size by Participant Characteristics **eTable 2.** Weighted Prevalence of Current Sole E-Cigarette, Dual (E-Cigarette and Combustible Cigarette), and Exclusive Combustible Cigarette Use Across Age Groups, 2021 Behavioral Risk Factor Surveillance System **eTable 3.** Age-Standardized Weighted Prevalence of E-Cigarette Use (Current and Daily) Across US States, 2019 Behavioral Risk Factor Surveillance System **eFigure 1**. Proportion of Current E-Cigarette Use Among Individuals With No Smoking History **eFigure 2.** Patterns of E-Cigarette and Combustible Cigarette Use by Age, 2021 Behavioral Risk Factor Surveillance System **eFigure 3.** Prevalence of Current E-Cigarette Use by State, 2021 Behavioral Risk Factor Surveillance System

Supplement 2.
Data Sharing Statement
