# Supplementary material for: E-Cigarette Use Among US Adults in the 2021 Behavioral Risk Factor Surveillance System Survey
Source: JAMA Netw Open. 2023 Nov 3;6(11):e2340859. doi: 10.1001/jamanetworkopen.2023.40859 (PMC10625038; doi:10.1001/jamanetworkopen.2023.40859)
Supplement: Supplement 1. — eTable 1. Weighted Sample Size by Participant Characteristics eTable 2. Weighted Prevalence of Current Sole E-Cigarette, Dual (E-Cigarette and Combustible Cigarette), and Exclusive Combustible Cigarette Use Across Age Groups, 2021 Behavioral Risk Factor Surveillance System eTable 3. Age-Standardized Weighted Prevalence of E-Cigarette Use (Current and Daily) Across US States, 2019 Behavioral Risk Factor Surveillance System eFigure 1. Proportion of Current E-Cigarette Use Among Individuals With No Smoking History eFigure 2. Patterns of E-Cigarette and Combustible Cigarette Use by Age, 2021 Behavioral Risk Factor Surveillance System eFigure 3. Prevalence of Current E-Cigarette Use by State, 2021 Behavioral Risk Factor Surveillance System [file jamanetwopen-e2340859-s001.pdf]

Supplementary Online Content

Erhabor J, Boakye E, Obisesan O, et al. E-cigarette use among US adults in the 2021 Behavioral Risk Factor Surveillance System survey. *JAMA Netw Open*. 2023;6(11):e2340859. doi:10.1001/jamanetworkopen.2023.40859

eTable 1. Weighted Sample Size by Participant Characteristics

eTable 2. Weighted Prevalence of Current Sole E-Cigarette, Dual (E-Cigarette and Combustible Cigarette), and Exclusive Combustible Cigarette Use Across Age Groups, 2021 Behavioral Risk Factor Surveillance System

eTable 3. Age-Standardized Weighted Prevalence of E-Cigarette Use (Current and Daily) Across US States, 2019 Behavioral Risk Factor Surveillance System

eFigure 1. Proportion of Current E-Cigarette Use Among Individuals With No Smoking History

eFigure 2. Patterns of E-Cigarette and Combustible Cigarette Use by Age, 2021 Behavioral Risk Factor Surveillance System

eFigure 3. Prevalence of Current E-Cigarette Use by State, 2021 Behavioral Risk Factor Surveillance System

This supplementary material has been provided by the authors to give readers additional information about their work.

© 2023 Erhabor J et al. *JAMA Network Open*.

eTable 1. Weighted Sample Size by Participant Characteristics

Variables	Weighted Sample Size	
	Current E-cigarette Use	Daily E-cigarette Use
**Total Population**	15,000,000	6,997,185
**Age**		
18-20	2,272,805	973,314
21-24	2,856,732	1,383,501
25-29	2,363,261	1,090,105
30-34	2,018,333	935,607
35-39	1,248,489	572,981
40-44	1,169,616	589,471
45-49	659,915	307,519
50-54	679,555	336,142
55-59	566,114	277,105
≥60	1,046,866	468,455
**Sex**		
Male	8,551,857	4,011,064
Female	6,453,806	2,986,121
**Race/Ethnicity**		
Non-Hispanic White	9,751,578	5,080,748
Non-Hispanic Black	1,344,852	461,156
Non-Hispanic Asian	728,114	220,823
Hispanic	2,231,529	806,769
NH or PI	60,566	30,449
AI or AN	171,728	57,754
Multiracial	354,166	161,164
Other	79,849	33,817
**Sexual orientation**		
Heterosexual	6,622,109	3,055,647
Lesbian/gay	311,718	173,790
Bisexual	853,060	443,441
Other	261,943	140,239
**Transgender**		
No	7,953,105	3,746,689
Yes	136,590	83,977
**BMI**		
<18.5kg/m^2	536,141	303,213
≥18.5kg/m^2 to <25/m^2	5,252,346	2,329,854
≥25/m^2 to <30kg/m^2	4,351,041	1,994,434
≥30 kg/m^2	4,182,311	2,036,613
**Marital status**		
Married	4,054,310	2,066,762
Divorced or separated	1,845,157	841,519
Widowed	359,701	181,468
Single	7,074,178	3,023,460
**Highest education level**		
Less than high school	1,747,529	755388
≥High school or some college	10,900,000	5,248,705
College graduate	2,356,639	981,171

© 2023 Erhabor J et al. *JAMA Network Open*.

Variables	Weighted Sample Size	
	**Current E-cigarette Use**	**Daily E-cigarette Use**
Income, poverty line, %		
<100	1,909,505	668,975
100-200	3,245,710	1,523,573
>200	9,740,794	4,746,371
**Employment status**		
Employed	9,823,671	4,855,126
Unemployed	2,963,902	1,293,731
Student	1,390,582	467,529
Retired	691,679	323,741
**Area of residence**		
Rural	945,570	461,091
Urban	6,511,678	6,511,678
**Combustible Cigarette Use**		
Never	4,882,539	1,720,260
Former	5,366,385	3,530,018
Current	4,632,683	1,690,674
**Pregnant**		
No	5,078,994	2,315,756
Yes	100,215	67,272
**CVD**[a]		
No	14,100,000	6,593,551
Yes	741,132	358,259
**Cancer**		
No	14,400,000	6,703,288
Yes	542,463	268,035
**COPD**		
No	13,900,000	6,508,483
Yes	1,037,259	268,035
**Asthma**		
No	11,800,000	5,473,904
Yes	3,128,123	1,473,624
**Depression**		
No	9,287,392	4,141,897
Yes	5,589,944	2,800,191

© 2023 Erhabor J et al. *JAMA Network Open*.

eTable 2. Weighted Prevalence of Current Sole E-Cigarette, Dual (E-Cigarette and Combustible Cigarette), and Exclusive Combustible Cigarette Use Across Age Groups, 2021 Behavioral Risk Factor Surveillance System

Age Groups, years	Weighted Prevalence, % (95% CI)		
	Exclusive E-cigarette Use	Exclusive Combustible Cigarette Use	Dual E-cigarette and Combustible Cigarette Use
**Total Population**	4.7 (4.6-4.9)	11.7 (11.4-11.9)	2.2 (2.1-2.3)
**18-20**	15.2 (13.9-16.7)	1.3 (1.0-1.8)	2.8 (2.3-3.3)
**21-24**	14.6 (13.6-15.6)	4.7 (4.1-5.4)	3.9 (3.4-4.4)
**25-29**	9.2 (8.5-10.0)	9.3 (8.6-10.0)	4.0 (3.5-4.6)
**30-34**	5.9 (5.1-6.4)	13.6 (12.8-14.5)	3.2 (2.9-3.6)
**35-39**	4.1 (3.7-4.6)	14.8 (14.0-15.6)	2.9 (2.6-3.3)
**40-44**	3.6 (3.1-4.0)	15.4 (14.6-16.2)	2.5 (2.1-2.9)
**45-49**	2.4 (2.1-2.7)	14.3 (13.4-15.3)	2.1 (1.7-2.5)
**50-54**	2.0 (1.7-2.4)	14.5 (13.7-15.3)	1.6 (1.4-1.9)
**55-59**	1.8 (1.6-2.1)	15.5 (14.7-16.3)	1.4 (1.2-1.6)
**≥60**	0.9 (0.8-1.0)	10.5 (10.1-10.8)	0.6 (0.5-0.7)

Note: Patterns are based on reported current use of e-cigarette and combustible cigarette use. Thus, sole e-cigarette use as defined may include individuals who formerly used combustible cigarettes

© 2023 Erhabor J et al. *JAMA Network Open*.

eTable 3. Age-Standardized Weighted Prevalence of E-Cigarette Use (Current and Daily) Across US States, 2019 Behavioral Risk Factor Surveillance System

States	Weighted Prevalence, % (95% CI)	
	Current E-cigarette Use	Daily E-cigarette Use
Alabama	9.1 (8.0-10.5)	4.4 (3.6-5.5)
Alaska	6.2 (5.2-7.3)	3.0 (2.3-3.8)
Arizona	8.8 (7.9-9.7)	4.3 (3.8-5.0)
Arkansas	7.3 (6.1-8.7)	4.2 (3.3-5.2)
California	5.2 (4.5-5.9)	2.2 (1.8-2.7)
Colorado	6.9 (6.3-7.5)	3.7 (3.2-4.2)
Connecticut	5.0 (4.3-5.8)	2.3 (1.8-2.9)
Delaware	6.1 (5.1-7.4)	2.9 (2.2-3.8)
District of Columbia	4.8 (3.6-6.3)	1.7 (1.0-3.0)
Georgia	7.8 (6.8-9.0)	3.6 (2.9-4.4)
Guam	11.1 (8.8-14.0)	5.7 (4.2-7.7)
Hawaii	7.3 (6.4-8.3)	3.3 (2.7-4.0)
Idaho	7.4 (6.5-6.3)	4.2 (3.5-4.9)
Illinois	6.4 (5.3-7.8)	3.2 (2.4-4.2)
Iowa	6.4 (5.7-7.2)	3.3 (2.8-3.9)
Indiana	8.1 (7.3-8.9)	4.0 (3.5-4.6)
Kansas	6.6 (6.1-7.2)	3.3 (3.0-3.7)
Kentucky	9.3 (8.2-10.5)	4.4 (3.6-5.3)
Louisiana	9.0 (7.8-10.3)	3.6 (2.9-4.5)
Maine	5.8 (5.1-6.7)	2.7 (2.2-3.4)
Maryland	4.5 (3.9-5.0)	1.8 (1.5-2.2)
Massachusetts	4.7 (4.1-5.4)	2.1 (1.7-2.6)
Michigan	7.6 (6.8-8.4)	3.3 (2.8-4.0)
Minnesota	5.7 (5.2-6.2)	2.6 (2.2-2.9)
Mississippi	6.4 (5.4-7.6)	2.9 (2.3-3.6)
Missouri	7.4 (6.6-8.2)	3.8 (3.3-4.4)
Montana	6.1 (5.2-7.0)	3.0 (2.4-3.8)
Nebraska	6.7 (6.1-7.4)	3.3 (2.9-3.9)
Nevada	7.0 (5.7-8.6)	2.6 (1.9-3.6)
New Hampshire	4.9 (4.1-6.0)	2.6 (2.0-3.5)
New Jersey	6.0 (5.2-6.8)	2.5 (2.0-3.0)
New Mexico	7.3 (6.3-8.3)	2.7 (2.1-3.4)
New York	5.3 (4.9-5.8)	2.4 (2.1-2.7)
North Carolina	6.8 (5.9-7.9)	3.3 (2.6-4.1)
North Dakota	7.4 (6.4-8.5)	3.7 (3.0-4.6)
Ohio	7.8 (7.1-8.6)	4.0 (3.5-4.6)
Oklahoma	9.4 (8.3-10.6)	4.6 (3.8-5.5)
Oregon	6.6 (5.8-7.5)	3.0 (2.4-3.6)

© 2023 Erhabor J et al. *JAMA Network Open*.

States	Weighted Prevalence, % (95% CI)	
	Current E-cigarette Use	Daily E-cigarette Use
**Pennsylvania**	6.1 (5.3-7.0)	2.8 (2.3-3.5)
**Puerto Rico**	2.0 (1.5-2.7)	0.7 (0.4-1.1)
**Rhode Island**	6.2 (5.1-7.4)	2.8 (2.1-3.6)
**South Carolina**	7.2 (6.3-8.2)	3.8 (3.2-4.5)
**South Dakota**	6.1 (4.9-7.7)	1.9 (1.4-2.7)
**Tennessee**	9.1 (8.0-10.3)	4.6 (3.8-5.6)
**Texas**	6.0 (5.1-7.0)	2.6 (2.1-3.3)
**Utah**	7.2 (6.5-7.9)	3.3 (2.8-3.8)
**Vermont**	5.0 (4.1-6.1)	2.6 (1.9-3.4)
**Virginia**	6.8 (6.0-7.7)	3.3 (2.8-4.0)
**Washington**	6.5 (5.9-7.2)	3.2 (2.8-3.7)
**West Virginia**	7.1 (6.3-8.1)	3.2 (2.6-3.9)
**Wisconsin**	6.2 (5.3-7.3)	3.0 (2.3-3.9)
**Wyoming**	7.5 (6.2-9.0)	4.1 (3.2-5.3)

© 2023 Erhabor J et al. *JAMA Network Open*.

eFigure 1. Proportion of Current E-Cigarette Use Among Individuals With No Smoking History

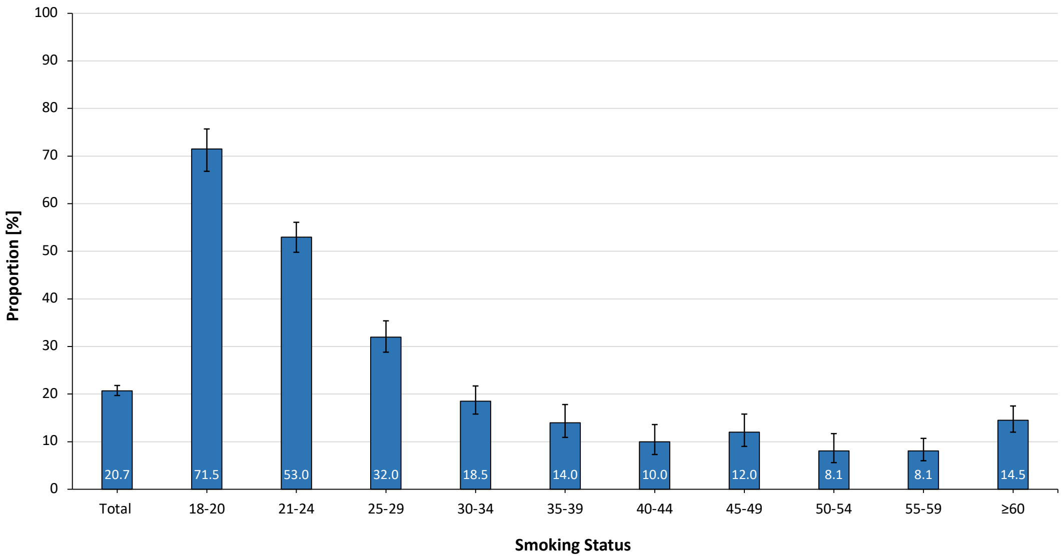

Error bars indicate 95% CIs

© 2023 Erhabor J et al. *JAMA Network Open*.

eFigure 2. Patterns of E-Cigarette and Combustible Cigarette Use by Age, 2021 Behavioral Risk Factor Surveillance System

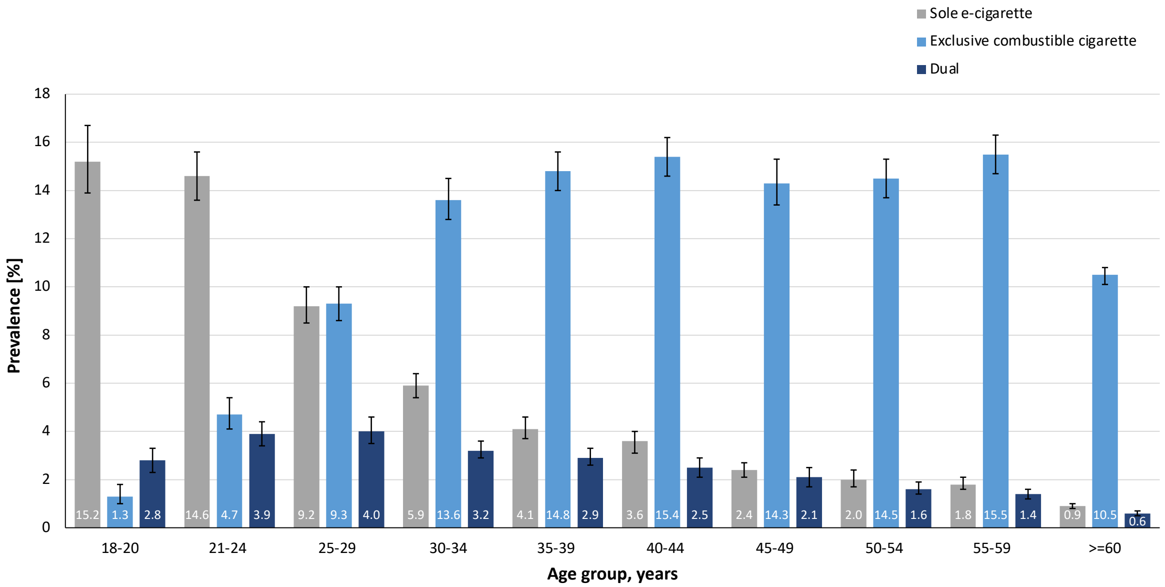

Error bars indicate 95% CIs. Sole e-cigarette use, as defined, may include individuals who formerly used combustible cigarettes, while dual use includes individuals who currently use both combustible cigarettes and e-cigarettes.

© 2023 Erhabor J et al. *JAMA Network Open*.

eFigure 3. Prevalence of Current E-Cigarette Use by State, 2021 Behavioral Risk Factor Surveillance System

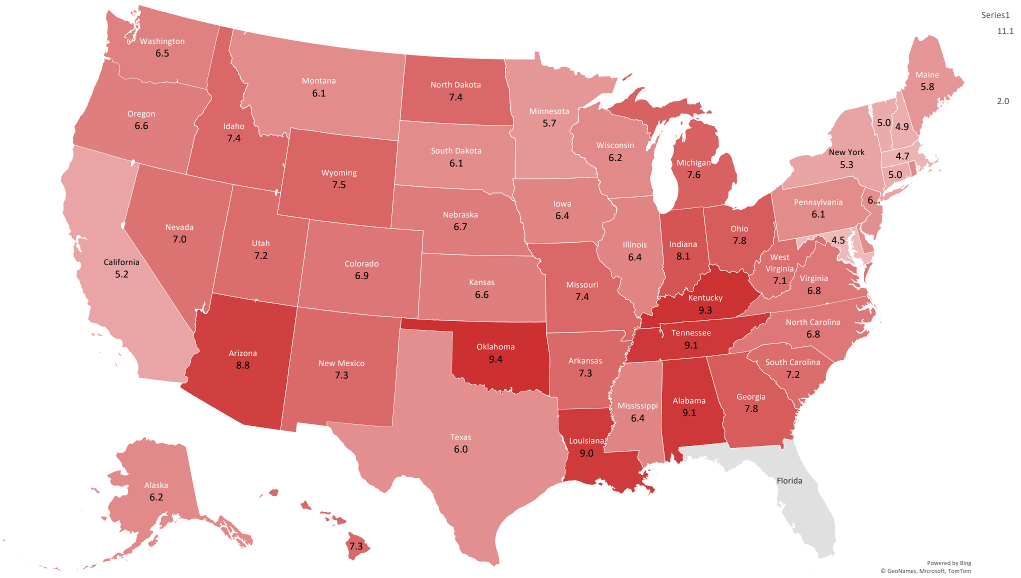

© 2023 Erhabor J et al. *JAMA Network Open*.
