# Supplementary material for: E-Cigarette Use Among US Adults in the 2021 Behavioral Risk Factor Surveillance System Survey
Source: JAMA Netw Open. 2023 Nov 3;6(11):e2340859. doi: 10.1001/jamanetworkopen.2023.40859 (PMC10625038; doi:10.1001/jamanetworkopen.2023.40859)
Supplement: Supplement 2. — Data Sharing Statement [file jamanetwopen-e2340859-s002.pdf]

Data Sharing Statement

Erhabor. E-Cigarette Use Among US Adults in the 2021 Behavioral Risk Factor Surveillance System Survey. *JAMA Netw Open*. Published November 03, 2023.
doi:10.1001/jamanetworkopen.2023.40859

Data
Data available: No

Additional Information
Explanation for why data not available: The BRFSS data are publicly available.
